# Development of Colonic Organoids Containing Enteric Nerves or Blood Vessels from Human Embryonic Stem Cells

**DOI:** 10.3390/cells9102209

**Published:** 2020-09-29

**Authors:** Chul Soon Park, Le Phuong Nguyen, Dongeun Yong

**Affiliations:** 1 Department of Laboratory Medicine and Research Institute of Bacterial Resistance, College of Medicine, Yonsei University, Seoul 03722, Korea; cspark0523@yuhs.ac (C.S.P.); luongphekidz07@gmail.com (L.P.N.); 2 Brain Korea 21 PLUS Project for Medical Science, Yonsei University, Seoul 03722, Korea

**Keywords:** human embryonic stem cell, organoid, colon, enteric nervous system, blood vessel

## Abstract

The increased interest in organoid research in recent years has contributed to an improved understanding of diseases that are currently untreatable. Various organoids, including kidney, brain, retina, liver, and spinal cord, have been successfully developed and serve as potential sources for regenerative medicine studies. However, the application of organoids has been limited by their lack of tissue components such as nerve and blood vessels that are essential to organ physiology. In this study, we used three-dimensional co-culture methods to develop colonic organoids that contained enteric nerves and blood vessels. The development of enteric nerves and blood vessels was confirmed phenotypically and genetically by the use of immunofluorescent staining and Western blotting. Colonic organoids that contain essential tissue components could serve as a useful model for the study of colon diseases and help to overcome current bottlenecks in colon disease research.

## 1. Introduction

Even though mice have been used as a disease model in biomedical fields, recent studies have shown that mice do not reproduce the gene expression patterns that are induced by inflammatory disease in humans. These studies have provoked a renewed discussion of the validity of animal models in translational research [1,2]. Organoids are well-organized structures that include various cell types from different developmental origins. Organoids containing structures found in whole organs have been generated from pluripotent stem cells via the developmental stages by use of three-dimensional (3D) culture systems [3]. An important advantage of organoids is that they display similar characteristics and morphology to human organs [4]. Although extensive research has been conducted on organoids, few studies exist of human colonic organoids (HCOs) [5,6]. The enteric nervous system (ENS) is implicated in a broad range of intestinal and extra-intestinal disorders [7]. Similarly, blood vessels play an important role in many diseases [8,9]. However, despite the central role of the ENS and blood vessels in intestinal function and health, our current understanding of their role is incomplete because of long-standing technical challenges in the development of these structures in organoid models [10].

Previously, Workman et al. described the methods for developing small intestine organoids containing enteric nervous system (ENS) [11], and Kasendra et al. developed human small intestine organoids from biopsy-derived stem cells [12]. For large intestine organoids, Múnera et al. developed colonic organoids from human pluripotent stem cells via BMP signaling activation [13]. Although methods of HCO development have been established over recent years, their lack of other structures such as nerves and blood vessels limits their applicability for the study of certain diseases such as ulcerative colitis, Crohn’s disease, and Hirschsprung’s disease. Ulcerative colitis is a chronic idiopathic inflammatory bowel disorder [14], and mucosal healing is a crucial factor for the treatment of refractory inflammatory bowel disease patients [15]. Even though a previous study indicated that endoscopy-assisted transplantation of intestinal stem cell plays as an emerging key role in the treatment [15,16], colonic organoids are much more important because the majority of ulcerative colitis lesions occurred in the rectum [17]. Moreover, transplantation of hematopoietic stem cells may improve the treatment outcome [18,19]. Besides, Crohn’s disease is also an inflammatory gastrointestinal with unknown etiology. However, in contrast to ulcerative colitis, this disease can affect both small and large intestine. On the other hand, Hirschsprung disease is a birth defect caused by the absence of neuronal ganglion nerve cells in different segments of bowel [20]. Previous study has engineered small intestinal organoids with ENS for Hirschspurng’s disease study [11]. Nevertheless, it was reported that 80% of patients had no neural ganglion in the rectosigmoid-segment and aganglionosis was rarely observed in the small intestine [21]. Accordingly, we developed HCOs that contain well-characterized enteric nerves or blood vessels, which is important for the investigation of colon diseases that involve these structures. Additionally, these HCOs can be used as more realistic predictive and prognostic models of colon diseases in humans.

## 2. Materials and Methods

### 2.1. Colonic Organoid Development Using Human Embryonic Stem Cells

**Figure 1:**
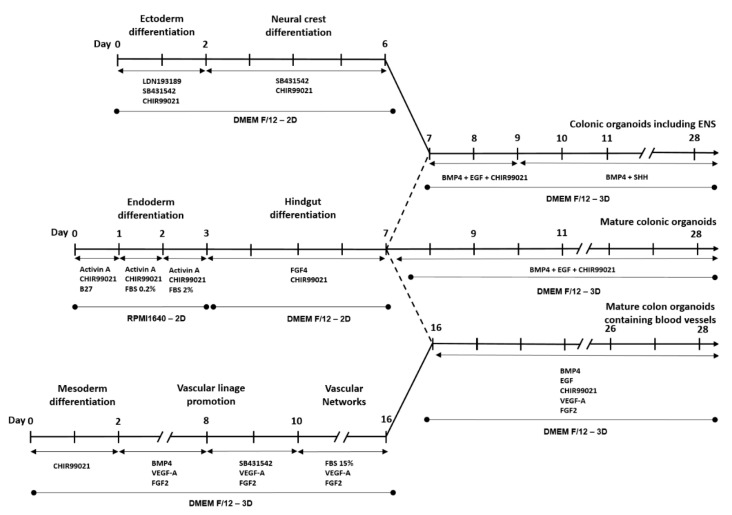
Timeline for the development of enteric nervous system (ENS) and blood vessel-containing human colonic organoids (HCOs) from human embryonic stem cells (hESCs). The dashed line indicates the 3D co-culture.

Human embryonic stem cells (hESC) (SNUhes31 cell line) were obtained from Seoul National University, Korea (Registration No. hES12010037) and incubated with 5% CO_2_ at 37 °C. The development of HCOs was performed in three distinct phrases including gendoderm and hindgut differentiation and colonic organoid development (Figure 1). Endoderm differentiation was performed in reference to the previous study with some modifications [22]. On day 1, 200,000 human embryonic stem cells (hESCs) were seeded in a 24-well plate and treated with B27 (1:100) (Gibco, Grand Island, NY, USA, 12587-010), Activin A (100 ng/mL, Peprotech, Rock Hill, NJ, USA, 120-14E), and mesoderm differentiation compound CHIR-99021 (3 µM, Sigma-Aldrich, St. Louis, MO, USA, SML1046-25MG), in which B27 is the key component to improve cell survival in definitive endoderm. RPMI1640 (Welgene, Gyeongsan, Korea, LM01101) was used as the cell culture media. On day 2, cells were treated with 0.2% fetal bovine serum (FBS) (Welgene, S001-07), Activin A (100 ng/mL), and CHIR-99021 (3 µM). On day 3, cells were treated with 2% FBS, Activin A (100 ng/mL), and CHIR-99021 (3 µM).

Hindgut differentiation was conducted according to Múnera et al. [13]. In brief, after 3 days of endoderm differentiation, cells were cultured for 4 days in Dulbecco’s Modified Eagle’s Medium (DMEM F/12) (Welgene, Gyeongsan, Korea, LM 002-04) supplemented with 2% FBS, fibroblast growth factor 4 (FGF4) (100 ng/mL, Peprotech, Rock Hill, NJ, USA, 100-31), and CHIR-99021 (3 µM).

### 2.2. Development of Colonic Organoids Containing Enteric Nervous System

Ectoderm differentiation was based on the protocol described by Jasan Tchieu et al. with some modifications [23]. In details, hESCs were cultured in DMEM F/12 with 10% Knockout Serum replacement (KSR) (Gibco, 10828-028). Ectoderm differentiation compounds, including LDN193189 (500 nmol, Sigma-Aldrich, St. Louis, MO, USA, SML0559), SB431542 (10 µM, Selleckchem, Houston, TX, USA, S1067), and CHIR-99021 (3 µM), were added for 2 days.

The development of HCOs containing enteric nerves was performed through treatment to promote ectoderm and neural crest differentiation and hindgut co-culture. Development of HCOs in Matrigel was performed according to previous study [13]. After 4 days of hindgut differentiation, Matrigel (BD Science, Franklin Lakes, NJ, USA, 354234) was used to 3D culture the cells, supplemented with BMP4 (10 ng/mL, R&D system, 314-BP-050), CHIR-99021 (3 µM), and EGF (500 ng/mL, Peprotech, AF-100-15). The cells were cultured in DMEM F/12 with B27. The development of complete colonic organoids required at least 28 days to 56 days in 3D culture.

Neural crest was also differentiated in reference to the previous study [23] with shorter time. In brief, ectoderm differentiated cells were cultured in DMEM F/12 medium supplemented with SB431542 (10 µM) and CHIR-99021 (3 µM) for 4 days.

In order to develop colonic organoid containing enteric nervous system, differentiated neural crest and hindgut co-culture was performed on Matrigel with BMP4 (10 ng/mL), CHIR-99021 (3 µM), and EGF (500 ng/mL) for 3 days. CHIR-99021 and EGF were then replaced by Sonic Hedgehog (SHH) (20 ng/mL, Peprotech, 100-45) for the remainder of the co-culture period.

### 2.3. Development of Colonic Organoids Containing Blood Vessels

Blood vessel development was performed according to previously described methods [24] with some modifications including mesoderm differentiation, vascular lineage promotion, vascular networks, and contact of the colon with vasculature. For mesoderm differentiation, hESCs were cultured for 2 days in DMEM F/12 with 20% KSR and CHIR-99021 (3 µM). Subsequently, for vascular lineage promotion, mesoderm-differentiated cells were treated with BMP4 (10 ng/mL), FGF2 (30 ng/mL, Sigma, SRP4037), and vascular endothelial growth factor A (VEGF-A) (30 ng/mL, Peprotech, 100-20) from day 3 to day 7. BMP4 was then replaced by SB31542 (10 µM) from day 8 to day 10. After that, cells were treated with 15% FBS, FGF2 (30 ng/mL), and VEGF-A from day 10 to day 16 for vascular network development. For the contact between the colon and vasculature, differentiated blood vessels were co-cultured with hindgut on Matrigel on day 17. The culture was then treated with BMP4 (10 ng/mL), EGF (500 ng/mL), CHIR-99021 (3 µM), and VEGF-A (30 ng/mL), FGF2 (30 ng/mL), then incubated until day 28. After that, subcultures were performed every 10 days for 56 days.

### 2.4. Colon Cancer Organoid Development

The colon cancer cell line SW480 (lot No. 7772) was obtained from Seoul National University (Seoul, Korea), and 3D culture was performed as described for HCO development.

### 2.5. Total RNA Extraction and Quantitative Reverse Transcription Polymerase Chain Reaction (qRT-PCR)

Total RNA extraction was performed using Qiazol (Qiagen, Hilden, Germany), chloroform (Duksan, Gyeonggi-do, Korea), and isopropanol (Sigma-Aldrich, St. Louis, MO, USA) according to previously described methods [25]. cDNA was synthesized using a high-capacity cDNA reverse transcription kit (Thermo Fisher, Waltham, MA, USA) according to the manufacturer’s instructions. Quantitative real-time (qRT)-PCR was performed using the KAPA SYBR^®^ FAST qPCR Master Mix (Sigma-Aldrich, St. Louis, MO, USA). The primer sequences used in this study are listed in Supplementary Materials, Table S1. All experiments were performed in technical triplicates.

### 2.6. Western Blotting

Western blotting was performed according to previously described methods, with some modifications [26]. In brief, 20 µg of each organoid lysate was separated on a 10% SDS-PAGE gel, then transferred onto a nitrocellulose membrane (GE Healthcare, Life Science, Chicago, IL, USA). The nitrocellulose membranes were blocked with phosphate-buffered saline with Tween 20 (PBST) buffer, then incubated overnight with primary antibody at 4 °C (Table S2). After washing, the membrane was incubated with goat anti-mouse mouse IgG antibody (H + L) (1:1000, Thermo Fisher, 31430). Clarity Western ECL Substrate (Bio-Rad, Hercules, CA, USA, 170-5060) with X-ray film was used to detect bands.

### 2.7. Hematoxylin & Eosin Staining

The organoids were removed from the gel state, then incubated overnight with 4% paraformaldehyde (PFA) solution at 4 °C. The staining was performed in the histopathology room at the Yonsei University College of Medicine (Seoul, Korea).

### 2.8. Immunofluorescent Staining

Immunofluorescent staining was performed according to previously described methods [13]. In brief, the organoids were slightly washed with 1X phosphate-buffered saline (PBS) solution, then fixed with 4% PFA (Sigma-Aldrich, MO, USA) for at least 1 h.

Washing, permeabilization, and blocking were performed for 30 min with bis-tris propane (BTP) buffer that included 0.5% bovine serum albumin (Genedepot, Katy, TX, USA, A0100-010) and 0.1% Triton X-100 (Sigma-Aldrich, St. Louis, MO, USA) in PBS. The primary antibody (1:500 to 1:1000) was incubated with organoids overnight at 4 °C (Table S2). The next day, organoids were washed with BTP buffer, then incubated with goat anti-mouse IgG (H + L) secondary antibody (1:1000, Thermo Fisher, 31430). Organoids were washed again with BTP buffer for 30 min, then incubated in with 4′,6-diamidino-2-phenylindole dihydrochloride (DAPI, 1 µg/mL) for 10 min at room temperature in dark. The organoids were observed on a fluorescence microscope after washing three times with 1X PBS over a 30-min period.

### 2.9. Statistical Analysis

The changes in the expression level for each gene were calculated as described by Livak et al. [27]. For each sample, the threshold cycle (Ct) of the target gene was determined and normalized to the Ct value of 18S rRNA and calculated relatively to the reference using the formula 2^−ΔΔCt^. The *p* values were determined using *t*-test in Prism GraphPad version 8.1.1.

## 3. Results

### 3.1. Development and Characterization of Colonic Organoids

**Figure 2:**
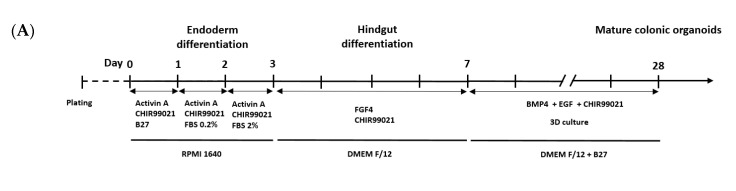

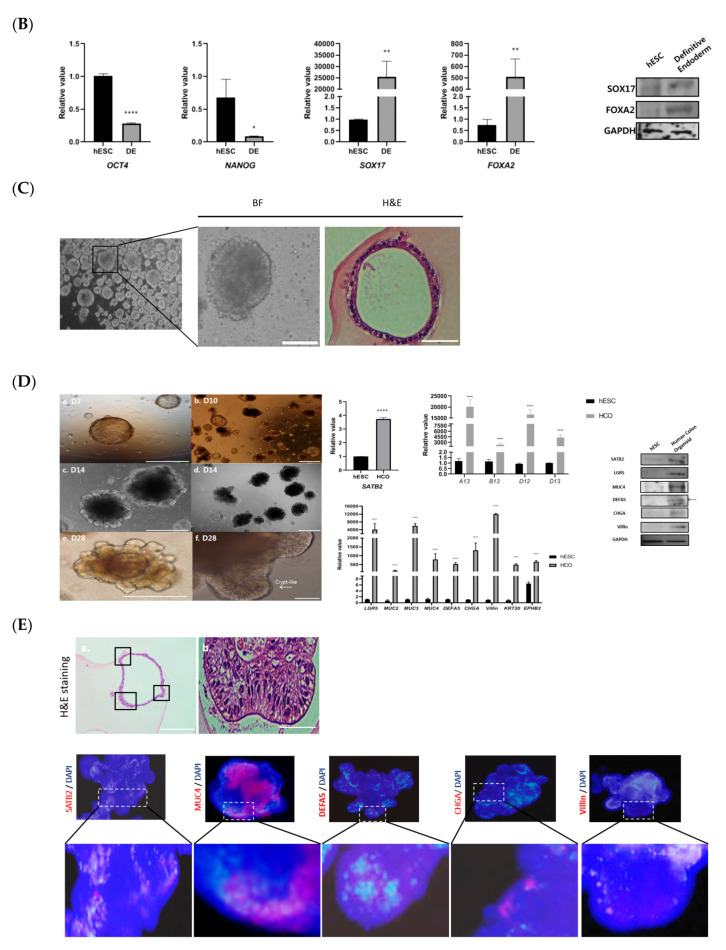
Development and characterization of HCOs. (**A**) HCO differentiation protocol. (**B**) Expression of pluripotency markers (*OCT4* and *NANOG*) and endoderm differentiation markers (*SOX17* and *FOXA2*) in the definitive endoderm (DE) of HCOs and untreated hESCs as detected via qRT-PCR. SOX17 and FOXA2 expression was detected by Western blotting, with GAPDH used as a loading control. (**C**) Hindgut (HG) morphology on bright-field (BF) microscopy and H&E staining. Expression of hindgut markers (*CDX2* and *KLF5*) as detected via qRT-PCR. (**D**) Development of morphologic changes in HCOs over time. Crypt-like structures had formed by day 28. Expression of colon-specific marker (*SATB2*); posterior *HOX* markers (*HOXA13*, *HOXB13*, *HOXD12*, and *HOXD13*); adult stem cell marker (*LGR5*); goblet cell markers (*MUC2*, *MUC3*, and *MUC4*); and markers for paneth cells (*DEFA5*), enteroendocrine cells (*CHGA*), and enterocytes (*villin*) by HCOs as detected via qRT-PCR. Detection of SATB2, LGR5, MUC4, DEFA5, CHGA, and villin by Western blotting, with GAPDH used as the loading control. The scale bars in panels (**a**–**e**) indicate 200 µm; the scale bar in panel (**f**) indicates 100 µm. (**E**): H&E and immunofluorescent staining of HCOs. The scale bar in panel (**a**) indicates 200 µm; the scale bar in panel (**b**) indicates 50 µm. * *p* ≤ 0.1, ** *p* ≤ 0.01, *** *p* ≤ 0.001, and **** *p* ≤ 0.0001. Scale bar: 200 µm.

Previous studies indicate that endoderm formation is characterized by decreased expression of pluripotency markers *OCT4* and *NANOG* [28] and increased expression of endoderm differentiation markers *SOX17* and *FOXA2* [29]. After 3 days of endoderm differentiation (Figure 2A,B), *OCT4* and *NANOG* expression levels were decreased in HCOs, compared with untreated hESCs. Conversely, *SOX17* and *FOXA2* were highly expressed in HCOs, compared with untreated hESCs. The expression of *SOX17* and *FOXA2* in HCOs was confirmed by Western blotting (Figure 2B).

Expression of *KLF5* and *CDX2* in hindgut differentiation has been previously demonstrated [30]. In the present study, after HCOs were treated with fibroblast growth factor 2 and CHIR99021 for 4 days, hindgut spheroids were observed on bright-field microscopy and on H&E staining. Increased expression of *KLF5* and *CDX2* in HCOs, compared with untreated hESCs, was confirmed via qRT-PCR and Western blotting (Figure 2C).

After at least 28 days of treatment with BMP4, CHIR-99021, and endothelial growth factor (EGF), the HCO morphology was observed to include crypt-like structures (Figure 2D). The expression levels of colon-specific marker (*SATB2*); posterior *HOX* markers (*HOXA13*, *HOXB13*, *HOXD12*, and *HOXD13*); adult stem cell marker (*LGR5*); goblet cell markers (*MUC2*, *MUC3*, and *MUC4*); and markers for paneth cells (*DEFA5*), enteroendocrine cells (*CHGA*), and enterocytes (*villin*), as detected by qRT-PCR, were increased in HCOs, compared with that in untreated hESCs (Figure 2D). This differential expression between HCOs and untreated hESCs was consistent with the results of a previous study [31]. The increased expression of SATB2, LGR5, MUC4, DEFA5, CHGA, and villin in HCOs was confirmed by Western blotting (Figure 2D) and immunofluorescent staining (Figure 2E). Furthermore, spherical hollow structures within the colon epithelium of HCOs were visible on H&E staining (Figure 2E).

The expression of markers and cells in colon cancer organoids (CCO) from colon cancer cell lines differed from that in HCOs (Figure S1). Compared with HCOs, the expression of *MUC4* and *villin* in CCOs was decreased, whereas there was no difference in the expression of *DEFA5* or *CHGA* between CCOs and HCOs.

### 3.2. Development of Colonic Organoids Containing Enteric Nervous System

**Figure 3:**
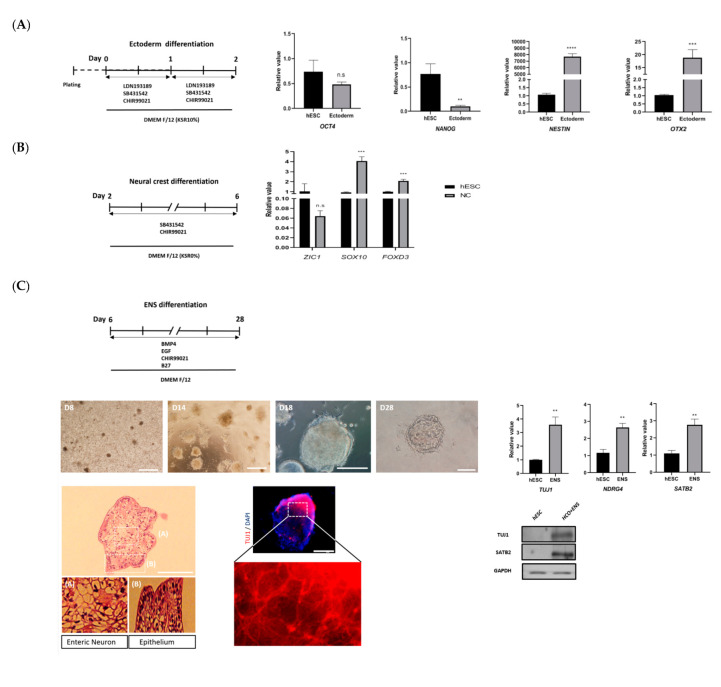
Development of HCOs containing ENS. (**A**) Ectoderm differentiation protocol and the expression of pluripotency markers (*OCT4* and *NANOG*) and ectoderm differentiation markers (*NESTIN* and *OTX2*) as detected via qRT-PCR. (**B**) Neural crest differentiation protocol and the expression of neural crest differentiation markers (*ZIC1*, *SOX10*, and *FOXD3*) as detected via qRT-PCR. (**C**) Development of HCOs with ENS development protocol. Morphology of HCO containing ENS at different time points. Detection of SATB2 and TUJ1 by Western blotting, with GAPDH used as the loading control. H&E staining of HCO containing ENS, and immunofluorescent staining of of TUJ1. Expression of neuron differentiation markers (*TUJ1* and *NDRG4*), and colon-specific marker (*SATB2*) as detected via qRT-PCR. ** *p* ≤ 0.01, *** *p* ≤ 0.001, and **** *p* ≤ 0.0001. n.s. indicates non significance. The scale bars indicate 200 µm.

Previous studies indicate that *Nestin* and *OTX2* are ectoderm differentiation markers [32], and *SOX10*, and *FOXD3* are markers for neural crest differentiation [33]. In addition, the expression of ectoderm and neural crest inhibitors such as *NANOG* and *OCT4* are reduced [34]. After 2 days of treatment with LDN193189, SB431542, and CHIR99021, there was a decrease in the expression of *NANOG* and *OCT4*, and the expression levels of *Nestin* and *OTX2* were increased in HCOs, compared with untreated hESCs (Figure 3A). The expression levels of *SOX10* and *FOXD3* were increased in HCOs, compared with untreated hESCs, after 4 days of treatment with LDN193189 and CHIR99021 (Figure 3B). Expression of the neural crest differentiation marker *ZIC1* did not increase, consistent with the findings of a previous study [35]. At day 28, the formation of HCOs together with enteric neurons was observed on bright-field microscopy and H&E staining (Figure 3C). The expression of the neuron differentiation markers *TUJ1* and *NDRG4* [35] indicated that enteric neurons successfully differentiated in conjunction with expression of SATB2 in both qRT-PCR and Western blotting (Figure 3C). Positive immunofluorescent staining for TUJ1 confirmed the successful formation of ENS and HCOs (Figure 3C).

### 3.3. Development of Colonic Organoids Containing Blood Vessels

**Figure 4:**
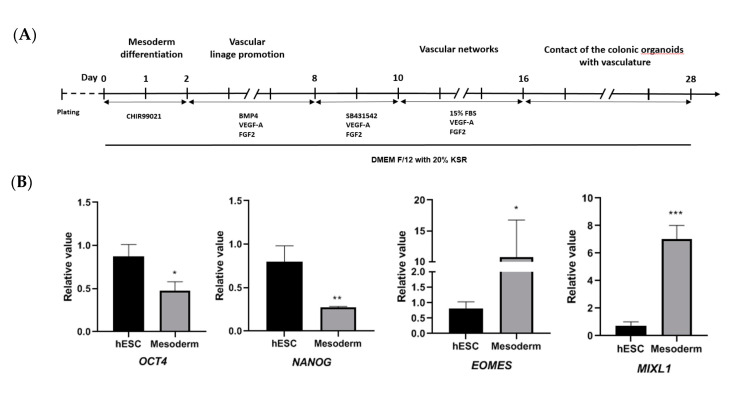

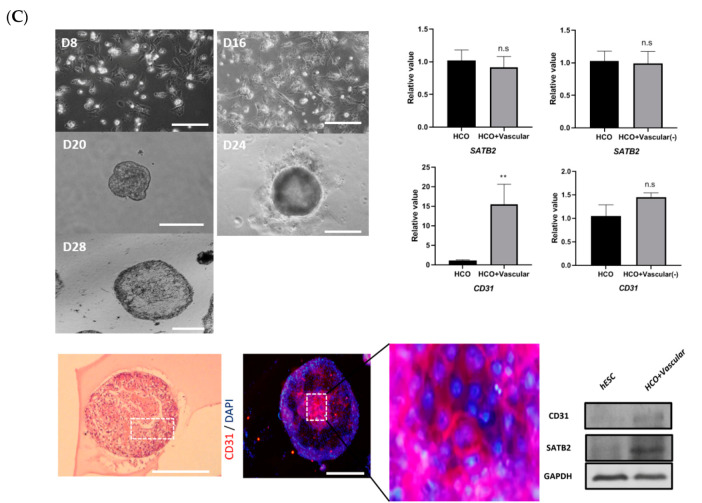
Development of HCOs containing blood vessels. (**A**) Timeline for development of blood vessel-containing HCOs. (**B**) Expression of pluripotency markers (*OCT4* and *NANOG*) and mesoderm differentiation markers (*EOMES* and *MIXL1*) as detected via qRT-PCR. (**C**) The morphology of blood vessel structures at days 8, 16, 20, 24, and 28. Immunofluorescent staining of blood vessel endothelial cell marker (CD31). Detection of SATB2 and CD31 by Western blotting, with GAPDH used as the loading control. H&E staining of HCO containing blood vessels, and immunofluorescent staining of CD31. Expression of colon-specific marker (*SATB2*) and blood vessel endothelial cell marker (*CD31*) as detected via qRT-PCR. n.s. indicates non significance. * *p* ≤ 0.1, ** *p* ≤ 0.01, and *** *p* ≤ 0.001. All the scale bars indicate 200 µm.

Initially, hESCs were differentiated into mesodermal cells by treatment with CHIR99021 for 2 days (Figure 4A). After CHIR99021 treatment, the expression of *OCT4* and *NANOG* was reduced and there was an increased expression of the mesoderm differentiation markers *EOMES* and *MIXL1* in HCOs, compared with untreated hESCs (Figure 4B). These results indicated successful mesoderm differentiation [28,36]. The vascular promotion stage, induced by treatment with BMP4, VEGF-A, and FGF4, was observed from day 2 to day 10. The vascular network formation stage, induced by treatment with SB43154, VEGF-A, and FGF4 from day 10 to 16, was confirmed on immunofluorescent staining (Figure 4C). Co-culture of blood vessels and hindgut was performed after day 16 by treatment with BMP4, EGF, CHIR-99021, VEGF-A, and FGF2. Blood vessels were formed with HCOs at day 20 (Figure 4C). The morphology of HCOs containing vessels at different time points was observed on bright-field microscopy and on H&E staining (day 28). Increased expression of the vascular marker CD31 [24] and HCO marker SATB2 in HCOs in both qRT-PCR and Western blotting, compared with untreated hESCs, indicated successful maintenance of the colon and vascular phenotype (Figure 4C). Successful formation of blood vessels in HCOs was confirmed by immunofluorescent staining of endothelial cell marker CD31 (Figure 4C). 

## 4. Discussion

Our protocol produced HCOs in a reasonably short period of time (28 days). All three germ layer types (endoderm, mesoderm, and ectoderm) were used to develop the ENS- and blood vessel-containing HCOs. We confirmed our results with qRT-PCR, Western blotting, and immunofluorescent staining. After co-culture of the ENS and HCOs, the expression of posterior HOX genes, including *HOXA13*, *HOXB13*, *HOXD12*, and *HOXD13*, was elevated in comparison with HCOs. These results were correlated with the previous study conducted by Múnera et al. [13] and suggested that the colonic morphology was well maintained after co-culture. However, in this study, hESCs were served as the original source for the development of colonic organoid; the application of adult stem cells has not been conducted yet. Further studies should be conducted to optimize the co-culture conditions of ENS, blood vessels, and colonic organoids derived from adult stem cells or induced pluripotent stem cells.

In addition, cancer organoids also play an important role in elucidating cancer biology, diagnostic, and prognostic markers [37]. Previous study has shown that cancer organoid models has more resemble gene expression profiles in vivo in comparison with 2D culture [38,39]. In this study, our group also successfully applied the current protocol for colon cancer organoids development from colon cancer cell line SW480. This model can be also served as a source for drug screening and biomarker testing. Furthermore, this procedure can be applied for the development of patient-derived organoids from adult stem cells, and facilitates the individualized anticancer drug sensitivity screening with more accurate prediction outcomes [37].

Additionally, further improvements are needed in which organoids are co-cultured with immune cells to create colonic organoids that more closely resemble the environment of the human colon. Recently, microfluidic technology and organoid-on-a-chip platforms have facilitated the development of multicellular structures with more consistencies in sizes and shapes of organoids [40]. Therefore, this protocol can also be applied to develop a more complex vessel-colonic organoids with immune cells.

Even though we successfully developed the ENS colonic organoids and vessels colonic organoids separately, the ENS and vessel-colonic organoids cannot be developed simultaneously. We tried to co-culture ENS and vessel organoids together but there are no morphological formation of HCOs containing ENS and vessels (Figure S2). Further study needs to be conducted to optimize the conditions for develop the more complicated organoids with both ENS and vessels.

## 5. Conclusions

In the present study, we successfully applied novel techniques for development of well-characterized enteric nerve and blood vessels in HCOs by 3Dculture. HCOs that contain the enteric nerves and blood vessels are important for improving our understanding of colon diseases and advancing research efforts in this area. This is especially true for the study of colon diseases that require the use of a model that closely resembles the human intestinal environment.

## Supplementary Materials

The following are available online at https://www.mdpi.com/2073-4409/9/10/2209/s1, Table S1: Sequences of primers used in this study, Table S2: Primary antibodies used in western blotting and immunofluorescent staining. Figure S1. Morphology of colon cancer cells (cell line SW480) and development of colon cancer organoids (CCO). Figure S2. Morphology HCOs using the triple co-culture of HCOs, ENS, and blood vessels.
