# Supplementary material for: Development of Colonic Organoids Containing Enteric Nerves or Blood Vessels from Human Embryonic Stem Cells
Source: Cells. 2020 Sep 29;9(10):2209. doi: 10.3390/cells9102209 (PMC7600593; doi:10.3390/cells9102209)
Supplement: Supplementary file 1 [file cells-09-02209-s001.pdf]

Table S1. Sequences of primers used in this study.

Genes	Forward (5′-3′)	Reverse (5′-3′)
18S	GGCCCTGTAATTGGAATG AG	GCTATTGGAGCTGGAATTAC
OCT4	GGGGTTCTATTTGGGAAGGTAT	TGTTGTCAGCTTCCTCCACC
NANOG	CAGAAGGCCTCAGCACCTAC	ATTGGAAGGTTCCCAGTCGG
SOX17	ACGCTTTCATGGTGTGGGCTAAG	GTCAGCGCCTTCCACGACTTG
FOXA2	CTTCAAGCACCTGCAGATTC	AGACCTGGATTTCACCGTGT
CDX2	ACACCTTCTACAATGAGC	ACGTCACACTTCATGATG
KLF5	GGTTGCACAAAAGTTTATAC	GGCTTGGCGCCCGTGTGCTTCC
HOXA13	AAATGTACTGCCCCAAAGAGCA	ATCCGAGGGATGGGAGACC
HOXB13	CCACTGGCTGCTGGACTGTT	TATGACTGGGCCAGGTTCTTTG
HOXD12	TGTGTGAGCGCAGTCTCTACAGA	CGGCCTCAGGTTGGAGAAG
HOXD13	CTGGGCTACGGCTACCACTTC	GCGATGACTTGAGCGCATT
SATB2	CCACCTTCCCAGCTTGATT	TTAGCCAGCTGGTGGAGACT
LGR5	CAGGGTCTTCACCTCCTACC	TGGGAATGTATGTCAGAGCG
MUC2	TGTAGGCATCGCTCTTCTCA	GACACCATCTACCTCACCCG
MUC3	CCTCATTGCAAACTTCACTC	AGCCCACATTTTCTGTACTG
MUC4	CGCGGTGGTGGAGGCGTTCTT	GAAGAATCCTGACAGCCTTCA
CHGA	CGGTTTTGAAGATGAACTCTCAG	GCTCTTCCACCGCCTCTT
DEFA5	ACCCAGAAGCAGTCTGGGAAGA	GGTGGCTCTTGCCTGAGAACCTGA
CHGA	CGGTTTTGAAGATGAACTCTCAG	GCTCTTCCACCGCCTCTT
VILLIN	TAGCTGTGGTTGTAAAGCAGTACC	GGTATCATCTTTCTGAAGGAATAGG
NESTIN	ATAGAGGGCAAAGTGGTAAGCAG	TTCTAGTGTCTCATGGCTCTGGTT
OTX2	GACCACTTCGGGTATGGACT	TGGACAAGGGATCTGACAGT
ZIC1	GTCCTACACGCATCCCAGTT	GTCCTACACGCATCCCAGTT
SOX10	CTCACTGCCCTGATGACCCA	CAGCCCCTCATCTTTCAGTGT
FOXD3	GACATGTTCGACAACGGCAG	CTGTAAGCGCCGAAGCTCTG
NDRG4	ATGCTTTCCATCCACTCACC	TTCACTGCTCTCTCCCGTTT
TUJ1	GAGGGAGATCGTGCACATCCAGG	CGAGTCGCCCACGTAGTTGC
EOMES	ATCATTACGAAACAGGGCAGG C	CGGGGTTGGTATTTGTGTAAGG
MIXL1	ACGTCTTTCAGCGCCGAACAG	TTGGTTCGGGCAGGCAGTTCA
BRACHY	GTGCTGTCCCAGGTGGCTTAC AGATG	CCTTAACAGCTCAACTCTAACTAC TTG
CD34	TACACGGAAAACGGTGGAGG	TTTTCTGAGCCCCTCGGTTC

Table S2. Primary antibodies used in western blotting and immunofluorescene staining.

Primary antibody	Catalog number	Company
SATB2	SC-81376	Santacruz
MUC4	SC-33654	Santacruz
DEFA5	SC-53997	Santacruz
VILLIN	SC-58897	Santacruz
CHGA	SC-393941	Santacruz
TUJ1	SC-51670	Santacruz
CD31	SC-376764	Santacruz
CD34	SC-7324	Santacruz
GAPDH	SC-47724	Santacruz
FOXA2	SC-374375	Santacruz
LGR5	MAB8078-SP	R&D System
SOX17	AF1924-SP	R&D System

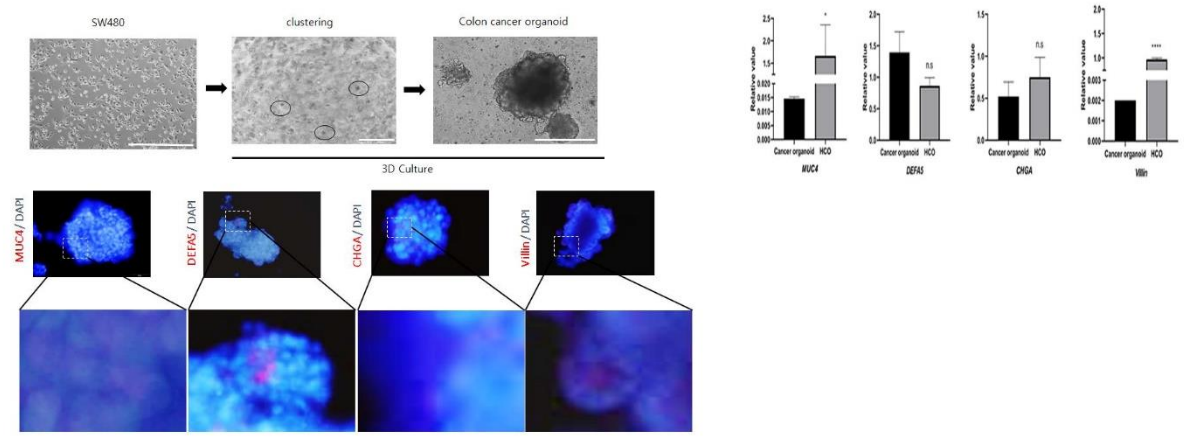

Figure S1. Morphology of colon cancer cells (cell line SW480) and development of colon cancer organoids (CCO). Expression of *MUC4*, *DEFA5*, *CHGA*, and *villin* of CCOs as detected via qRT-PCR and immunofluorescent staining. *p ≤ 0.1, **p ≤ 0.01, ***p ≤ 0.001, and ****p ≤ 0.0001. Scale bar, 200 μm.

A.   B.

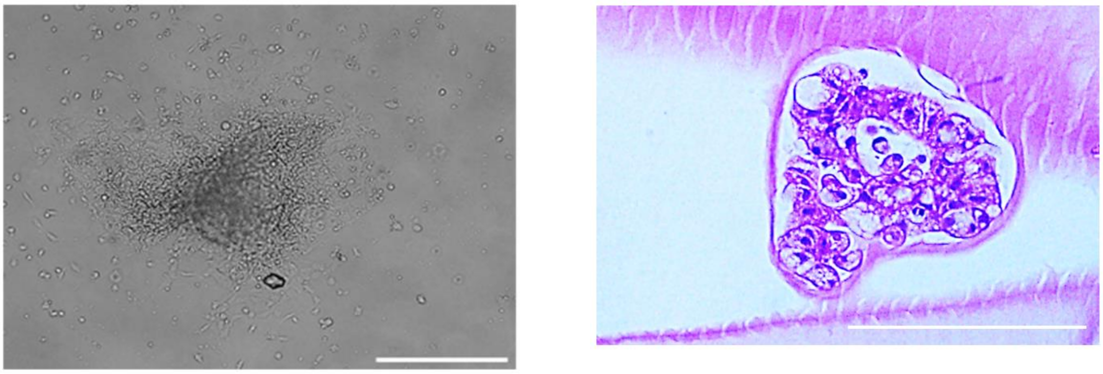

Figure S2. Morphology HCOs using the triple co-culture of HCOs, ENS and blood vessels. (A): Brigh-field microscopy of HCOs after 7 days of triple co-culture. (B): H&E staining of HCOs after 7 days of triple co-culture. The scale bars indicate 200 μm.
